# Habituation to a predatory stimulus in a harvester (Arachnida, Opiliones)

**DOI:** 10.1007/s10071-024-01857-7

**Published:** 2024-03-05

**Authors:** Guilherme Ferreira Pagoti, Jerry A. Hogan, Rodrigo Hirata Willemart

**Affiliations:** 1 Laboratório de Ecologia Sensorial e Comportamento de Artrópodes, Escola de Artes, Ciências e Humanidades, Universidade de São Paulo, Rua Arlindo Béttio, 1000, Ermelino Matarazzo, São Paulo, SP 03828-000 Brazil; 2 Programa de Pós-Graduação em Zoologia, Instituto de Biociências, Universidade de São Paulo, Rua do Matão, 321, Travessa 14, Cidade Universitária, São Paulo, SP 05508-090 Brazil; 3 Department of Psychology, University of Toronto, 100 St. George Street, 4th Floor Sidney Smith Hall, Toronto, Ontario M5S 3G3 Canada

**Keywords:** Defensive behavior, Experience, Non-associative learning, Retention

## Abstract

Several studies have investigated habituation in a defensive context, but few have addressed responses to dangerous stimuli. In such cases, animals should not habituate since this could cost their lives. Here we have stimulated individuals of the harvester *Mischonyx squalidus* with a predatory stimulus (squeezing with tweezers) in repeated trials within and between days, and measured the occurrence and magnitude of nipping, a defensive behavior. Contrary to our expectations, they did habituate to this stimulus. The probability and magnitude of response declined over trials during each of three days of testing in a typical habituation pattern. During the trials we also observed other defensive behaviors. We discuss our results mainly considering alternative defensive responses. Our data show that we lack information on (1) the role played by the ambiguity of stimuli, (2) the role played by subsequent stimuli and (3) the importance of the array of defensive behaviors of a species in understanding habituation. Although ubiquitous across animals and therefore expected, habituation is described for the first time in the order Opiliones.

## Introduction

Habituation is a non-associative learning process defined as a decrease of a response that results from the repetition of a stimulus if there is no motor or sensory fatigue (Rankin et al. 2009). It is important because it allows animals to distinguish between irrelevant and relevant stimuli, saving energy by ceasing responding to the former (Eisenstein et al. 2001; Bell and Peeke 2012). We have been particularly interested in how some stimuli may be ambiguous from the perspective of the prey (Liang et al. 2019). To respond optimally to stimuli, animals require reliable information and should respond differently to ambiguous and to actual high-risk predatory stimuli (e.g., Raderschall et al. 2011; Wolfe et al. 2016). Responding repeatedly to harmless shadows, for example, would probably deplete energy reserves (Rodríguez-Prieto et al. 2006). This distinction becomes even more important when the effects of habituation last for more than 24 h (Rankin et al. 2009).

If animals can remember information about a dangerous event, such learning may positively influence future behavior (Shettleworth 2010). After experiencing a predatory attack, being able to maintain a long-term memory could improve not only the defensive behavior itself, but also help avoiding future encounters with such predators by using cues released by the predator in the environment (Pueta et al. 2021).

When facing unambiguously dangerous stimuli, animals should not habituate since this could cost them their lives. Different non-associative learning theories could help understanding, at least mechanistically, those situations, such as sensitization or the dual-process theory in cases where the stimulus is highly arousing/sensitizing (Groves and Thompson 1970; Eisenstein et al 2001). However, regardless of the explanation, the unambiguity of the stimulus per se has not been specifically addressed or studied, that is, there has been no comparison of reactions towards ambiguous and unambiguous stimuli. Previous papers that have reported the absence of habituation (and/or sensitization) are cases in which the stimuli used happened to be unambiguous (Prestrude and Crawford 1970; Zangrossi and File 1992; Hemmi and Zeil 2003; Masini et al. 2006; but see: Pueta et al. 2021).

Another important factor is the level of threat imposed by a predatory stimulus. According to the threat-sensitive hypothesis, animals are expected to modulate their defensive response according to how dangerous the stimulus is (Helfman 1989). Animals are expected to adjust their antipredator behavior to match the actual level of predation risk, therefore avoiding the cost of unnecessary predator avoidance behaviors (Rodriguez-Pietro et al. 2010).

Both short-term and long-term habituation have been reported with unambiguous stimuli (predator model at a distance and predator sound (Long et al. 1989; May and Hoy 1991)) but both these stimuli are at the “less dangerous” end of the continuum. In contrast, being handled by the predator resides at the opposite and most threatening end of the continuum.

Here we looked at habituation to an unambiguous stimulus that is highly dangerous to the prey. We therefore expected to observe no short-term habituation and that consequently there would be no long-term habituation of the behavior involved. We tested this hypothesis using the armored harvester *Mischonyx squalidus* (Roewer, 1913) (Arachnida, Opiliones). To our knowledge, this is the first study to investigate habituation and long-term habituation in the order Opiliones using a predatory context.

## Material and methods

### (a) Study animal, collection, and maintenance

*Mischonyx squalidus* appears in previous articles as *Mischonyx cuspidatus* or *Ilhaia cuspidata* (see Gueratto et al. 2021). We collected 25 adult males of *M. squalidus* at the Parque Ecológico do Tietê (−23.507722, −46.547899), in the city and State of São Paulo in July 2022. We maintained them in individual terraria (10 × 5 × 5 cm height) and fed them twice a week with damp dog food. Water was provided in a damp cotton ball. We kept the room temperature at 24º C and the light was ambient.

### (b) Eliciting nipping behavior

Nipping is a well-known defensive behavior in harvesters. Mainly males can nip an aggressor by rapidly flexing both legs IV. Particularly in *Mischonyx squalidus*, nipping has been documented to pierce, cut, and repel predators (Dias and Willemart 2013; Segovia et al. 2015; Silva et al. 2018) and pierce human skin (GFP personal observation). This behavior can be reliably elicited by holding the harvester´s body dorso-ventrally with tweezers, a procedure that mimics capture by a bird (Gnaspini and Hara 2007 and references therein). Tweezers also allow control of the force applied and can be compared with the actions of birds, a known predator of harvesters (Cokendolpher and Mitov 2007).

In our experiments, we have used tweezers with a drop of melted hot glue on its tips to avoid damaging the animal´s cuticle. To minimize differences of strength between trials, we used a peg connected to the tweezers to apply a pressure of 20 N (measured with a dynamometer). This pressure is in the range of the biting force of several birds (Herrel et al. 2005). Moreover, because in nature only a predator would seize, squeeze and lift the harvester off the ground, we considered our stimulus a fair simulation of a predator. If there was no response after the first stimulus, we reapplied it two more times and the test resumed after the first nipping. We applied 10 sequential stimuli with an interval of 3 s between each stimulus. This interval time was chosen from a previous experiment for another study, in which we have observed hens biting harvesters repeatedly with intervals of 2 or 3 s (GFP personal observation). To test for dishabituation, we used an 11th stimulus that consisted of stimulating the chelicerae with a brush (that usually causes the harvester to respond by trying to grab the brush with the chelicerae and/or pedipalps) and after 3 s we applied the regular pressure stimulus again. To test for long-term habituation, we repeated the exact same procedure with the same animals 24 h and 48 h after the first test, making ten trials (plus one dishabituation trial) on each of the 3 days (n = 25 males).

**Fig. 1:**
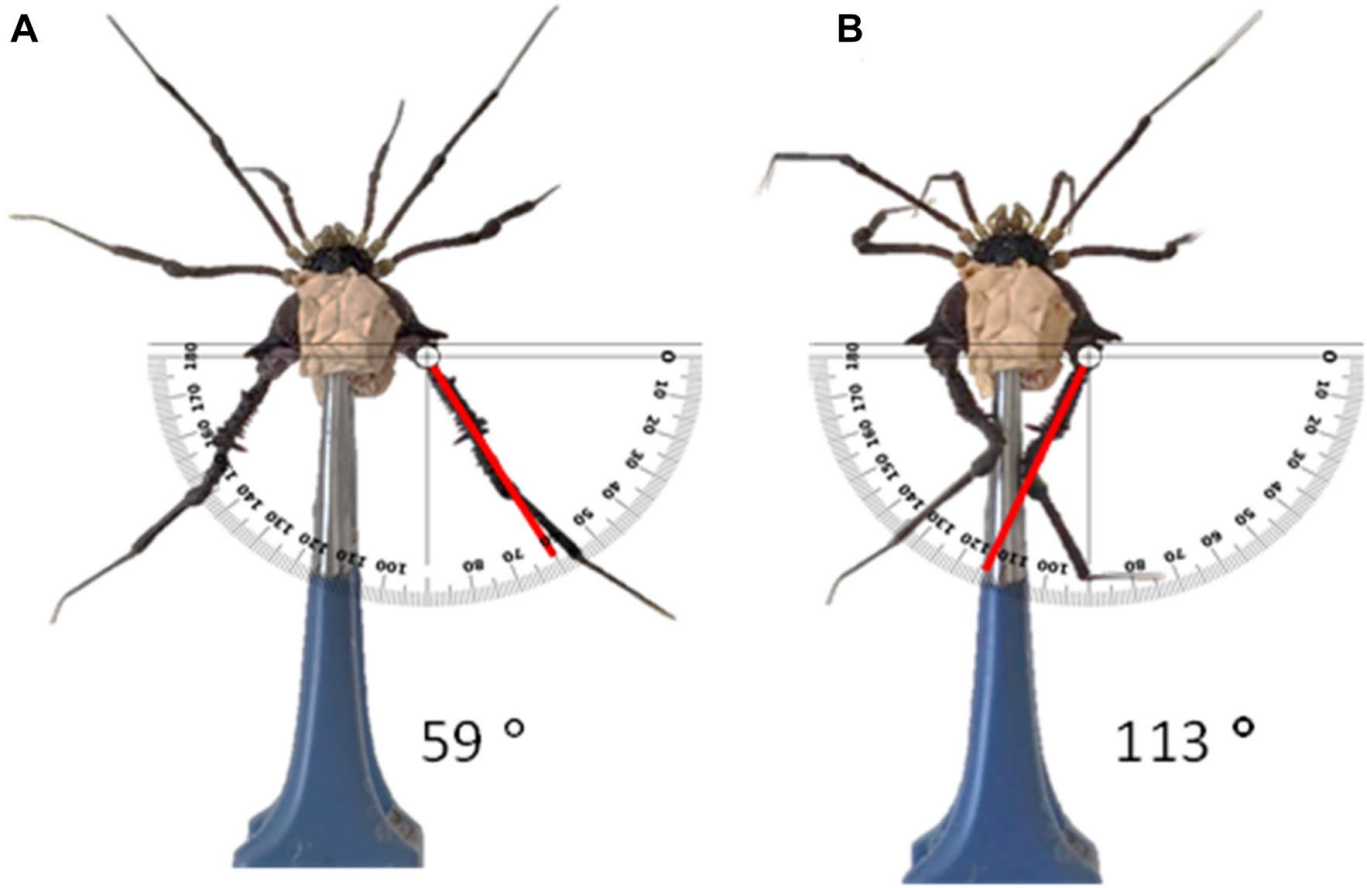
Method using tweezers for triggering nipping with legs IV in the harvester *Mischonyx squalidus*. The initial angle of 59° is shown on **A** and the final angle of 113° is shown on **B**

We noted if the animals nipped (flexing the legs so that the coxa-trochanter area articulates) or did not nip (legs motionless) and we also checked the videos for the magnitude of the behavior, looking at the difference between the angles of the legs IV before and after the nipping movement (Fig. 1). The nipping behavior is a quick (usually 1 s or less of duration) pinching movement that causes the spined femur of both legs IV to cross each other, hitting the target between the legs (Fig. 1B). After pinching, the legs return to their normal posture (Fig. 1A). The animal can perform several nipping behaviors in a sequence. During the tests we also scored other defensive behaviors that can be elicited with repeated stimulation (Rankin et al. 2009).

### Statistical analyses

To analyze the responses during trials, days, and possible interaction between the two, we applied a generalized linear mixed model (GLMM, package lme4) with a binomial structure (logit-link). All the response variables were either 1or 0 (if the responses occurred or not, respectively). Days and trials were included as fixed effects and individual ID as a random effect to account for repeated measures. To analyze the dishabituation phase, we applied a McNemar’s test between trial 10 and trial 11 (dishabituation) for each day.

We also measured the magnitude of response of the nipping behavior as the difference between the angles of the legs IV before and after the nipping movement. To analyze that response during trials, days, and possible interaction between the two we also applied a generalized linear mixed model (GLMM, package glmmTMB) with Gamma structure for inflated-zero model, to guarantee that all the trials and responses were included in the analysis. Days and trials were included as fixed effects and individual ID as a random effect to account for repeated measures. All statistical analyses were performed using ‘‘R” software, version 3.5.3 (www.r-project.org; R Development Core Team, 2008)**.**

## Results

**Fig. 2:**
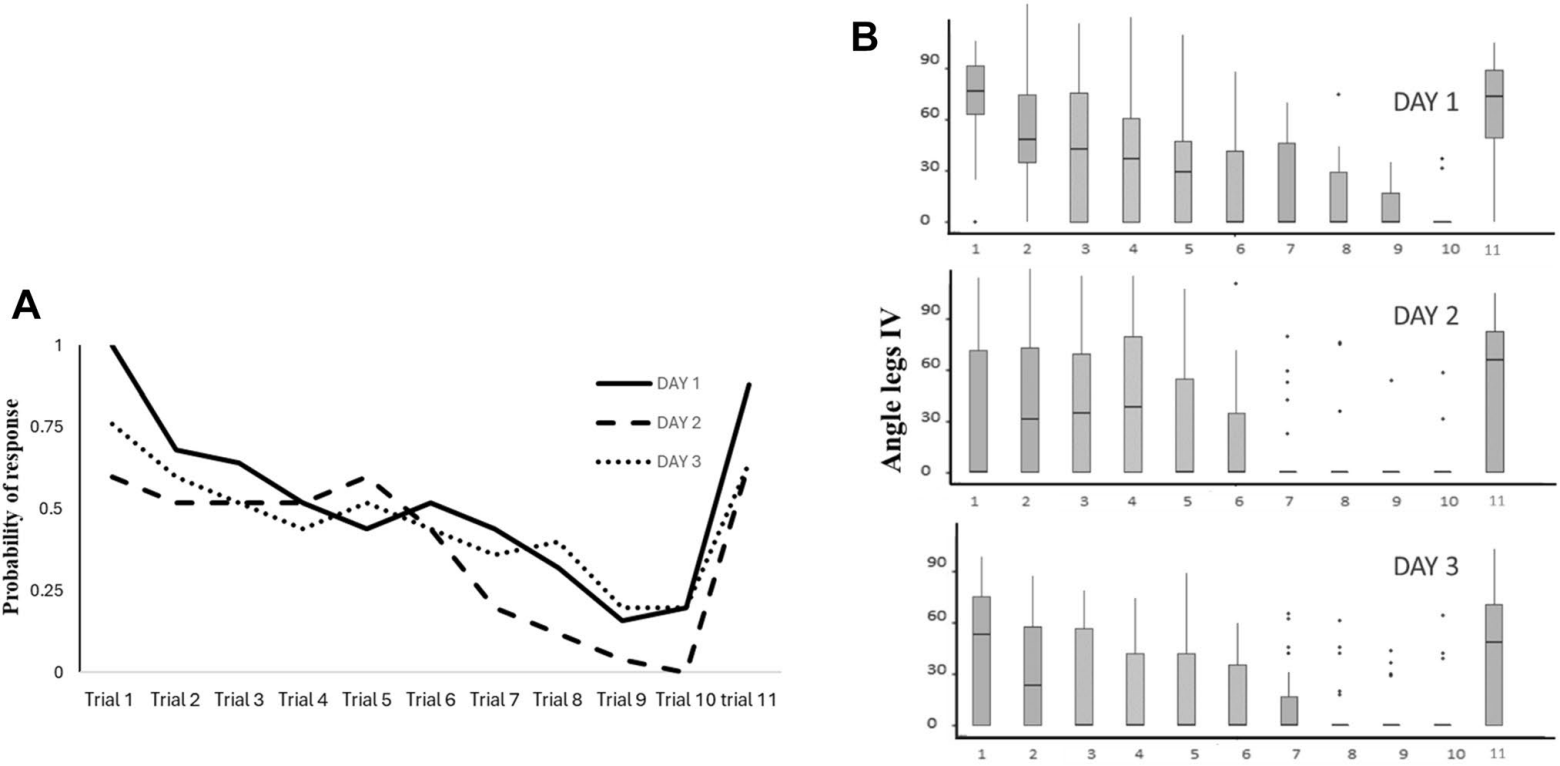
**A** Probability of delivering a nip after being stimulated by tweezers in the harvester *Mischonyx squalidus*, per trial and per day. **B** Box plot of the magnitude of response. The angles are the difference between the same leg IV before and after being stimulated by tweezers in the harvester *M. squalidus*. Trial 11 is showing the dishabituation trial on both graphics

The results for the habituation trials are shown in Fig. 2. The probability and magnitude of response declined over trials each day in a typical habituation pattern, and overall responding was lower on the second and third days of testing. For the probability of responding to the stimulus, we found differences between trials (GLMM, CI = 0.46–0.67; z = −6.043; p < 0.001), and between days (GLMM, CI = 0.34–0.92; z = −2.298; p < 0.022), but no positive interaction between days/trials, (GLMM, CI = 1.00–1.18; z = 1.879; p = 0.060).

The results for the magnitude of response are also similar. We found differences between trials (GLMM, CI 1.24–1.83; z = −5.740; p < 0.001) and days (GLMM, CI = 1.60–4.83; z = 3.621; p < 0.001) but no positive interaction between trial/day (GLMM, CI = 0.86–1.02; z = −1.399; p = 0.162).

The stimulus used for dishabituation (trial 11) elicited more intense responses than in the last trial of each day for magnitude and probability. For magnitude, on the first day the median of the angle between legs IV on trial 11 was 65º (max = 93º; min = 0º) while in trial 10 it was 0º (max = 33º; min = 0º). On the second day the median of the angle between legs IV on trial 11 was 59º (max = 94º; min = 0º) and for trial 10 the median was 0º (max = 52º; min = 0º). Finally, on the third day the median of the angle between legs IV on trial 11 was 44º (max = 93º; min = 0º) and for trial 10 the median was 0º (max = 52º; min = 0º). We also found differences in the probability of response. On day 1 the probability of response on trial 11 was 88% and for trial 10 it was 2% (X^2^ = 7.1; df = 1; p = 0.007). On the second day the probability of response on trial 11 was 64% and for trial 10 it was 0% (X^2^ = 7.1 = df = 1; p = 0.007). Finally on the third day the probability of response on trial 11 was 64% and for trial 10 it was 20% (X^2^ = 12; df = 1; p = 0.005). These disinhibition tests suggest that the animals did not decrease their response due to sensory or motor fatigue.

We also observed another defensive behavior, namely the release of chemicals (n = 16 in 750 observations), a typical defense response in harvesters (Gnaspini and Hara 2007). Most of these observations (n = 14) happened on the first day of trials and the other two on the second day. They all occurred after the fifth trial and only a single animal released the chemical twice (on different days). Although this strategy occurred infrequently (only about 2% of the observations), it was used by more than 50% of the animals (15 of 25) across the experiment. We could not detect any specific causal agent that triggered such behavior. We also observed two cases of thanatosis (playing dead, cf. Segovia et al. 2015).

## Discussion

We have shown that habituation of a defensive response occurs to an unambiguously threatening stimulus in individuals of *M. squalidus.* Animals also showed a decrease in their responsiveness, but with a similar habituation pattern, over consecutive days. We used a highly threatening stimulus that resembled a predator, since no stimulus other than a predator would pinch and lift the harvester´s body. Moreover, as explained above, we applied a force similar to a bird bite, although we did not provide other putative relevant sensory cues such as shade or olfactory cues of a predator (see Pueta et al. 2021). We were not expecting habituation to occur based on examples from previous work (Prestrude and Crawford 1970; Zangrossi and File 1992; Masini et al. 2006; Ardiel et al. 2017; Pueta et al. 2021).

Proximately, it has been shown that repeated stimulation of specific nociceptive neurons decreases the magnitude of a given response, causing habituation (Ardiel et al. 2016). However, repeated activation of such neurons may also have other effects, including behavioral responses. For example, other defensive strategies could be evoked (Ardiel et al. 2017; McDiarmid et al. 2019). The use of defensive mechanisms other than nipping might be associated with a switching of the defensive strategy. This might be a result of habituation in one defensive mechanism and sensitization in another defensive mechanism (Rushford et al. 1963; Evans 1969; Ardiel et al. 2017). It is noteworthy that the release of chemicals, when it occurred, happened after the animals had stopped responding with nipping. The composition of this defensive chemical is a blend of several components, including benzoquinones and phenols among others (for more details see Hara et al. 2005). The release of this compound, though efficient (Machado et al. 2005; Silva et al. 2018), is costly (Nazareth and Machado 2015; Nazareth et al. 2016), which may justify why none of the animals released chemicals more than once on the same day.

Another interesting point is that animals have been reported to habituate the magnitude of the response but not the probability of responding (Ardiel et al. 2017). However, our data show habituation in both variables. Because the defensive nipping did not prevent the stimulus from occurring, we can only speculate that the animal would stop reacting with nipping and instead rely on other defensive mechanisms were it subsequently manipulated in the predator’s mouth. In such a case, heavy armor, spines, or chemical defenses would serve this purpose (see references in Gnaspini and Hara 2007; Silva et al. 2018).

We also found a difference in the probability of responsiveness on successive days. Individual differences are indeed expected according to the Behavioral Homeostasis Model (Eisenstein et al. 2001) since some individuals have lower sensory thresholds. However, our sample size does not allow us to make any solid statement about whether or not we found support for this model, which predicts differences between individuals and a relation between sensory thresholds and responsiveness to stimuli, sensitization and habitation. Still, the animals in our study might have learned that the stimulus is not dangerous because (1) both the context and the inter-stimulus interval (ISI) were precisely the same (see McDiarmid et al. 2019), which would be unusual in nature (May and Hoy 1991) and (2) although the harvesters were seized, lifted and squeezed, they were apparently not harmed. Furthermore, the interaction between trials/days was not significant. The general habituation pattern was similar between days, but on days 2 and 3 there was a slightly lower probability of responses, compared to day 1 (Fig. 2). In long-term habituation, the responses typically do not return to naïve levels (Tomsic et al. 1993; Rankin et al. 2009; Randlett et al. 2019). Moreover, the maintenance of this pattern of response with higher probability of response in the first trials may be an active process of not ignoring the stimulus at all. This may be important to trigger the possibility of switching to another defensive strategy, which could be of high survival value when facing potential lethal stimuli (Ardiel et al. 2017; McDiarmid et al. 2019). Learning by habituation might be an optimization of behavioral strategies in accordance with particular internal (physiological) and external (environmental context) factors.

In summary, we have shown that habituation occurs following a dangerous stimulus and that memory follows in this context. Our study suggests we need a better understanding of the roles of ambiguity of the stimulus, the influence of how the animal responds to subsequent behaviors, and the specific role of each defensive behavior. We also present a new technique to test habituation and dishabituation in a new model organism (Opiliones).

## Data Availability

Data supporting this study are included within the article and/or supporting materials.
